# First description of *Hepatozoon canis* in raccoon dog (*Nyctereutes procyonoides*)

**DOI:** 10.1016/j.ijppaw.2025.101132

**Published:** 2025-09-02

**Authors:** Itainara Taili, Jongseung Kim, Sungryong Kim, Dong-Hyuk Jeong, Ki-Jeong Na

**Affiliations:** a Laboratory of Veterinary Laboratory Medicine, College of Veterinary Medicine, Chungbuk National University, Cheongju, 28644, South Korea; b Laboratory of Wildlife and Conservation Medicine, College of Veterinary Medicine, Chungbuk National University, Cheongju, 28644, South Korea; c The Wildlife Center of Chungbuk, Cheongju, 28116, South Korea

**Keywords:** Hepatozoon, One health, PCR, Raccoon dog, Wildlife

## Abstract

*Hepatozoon canis* is a tick-borne apicomplexan parasite that primarily infects domestic and wild canids. While its presence has been documented globally, previous studies have reported its absence in raccoon dogs (*Nyctereutes procyonoides*) in Europe, and its status in Asian populations remains unclear. This study presents the first molecular detection of *H. canis* in raccoon dogs in South Korea. Between 2021 and 2023, blood samples from 275 raccoon dogs admitted to 9 wildlife centers were analyzed using PCR targeting a partial sequence of the 18S rRNA gene. Overall, 21.5 % of samples tested positive, with the highest prevalence observed in the southern region (38.2 %) and the lowest in the north (8.8 %) in South Korea. Sequencing of amplicons revealed high similarity to *H. canis* found in a Japanese hard tick (*Ixodes nipponensis*) also from South Korea. Remarkably, the infection rate in raccoon dogs was significantly higher than previously reported in Korean domestic dogs (0.2–0.9 %) and ticks (0.09 %), indicating raccoon dogs may function as key sylvatic reservoirs. These findings suggest the possibility of alternative transmission pathways including predation or vertical transmission. Given the expanding raccoon dog population and thus increasing contact with domestic animals and shared habitats, their role in the ecology of *H. canis* and other tick-borne pathogens merits attention. This study underscores the importance of wildlife disease surveillance within the One Health framework and highlights the need for further research into host–vector dynamics and potential spillover risks at the wildlife–domestic animal interface.

## Highlights

• First molecular evidence of *Hepatozoon canis* in raccoon dogs (Nyctereutes procyonoides).
• 21.5 % of 275 raccoon dogs in South Korea tested positive by PCR.
• *Hepatozoon canis* prevalence in raccoon higher than in Korean domestic dogs.
• Raccoon dogs may become key sylvatic hosts for *H. canis* in native ecosystems.

## Graphical abstract

**Figure:**
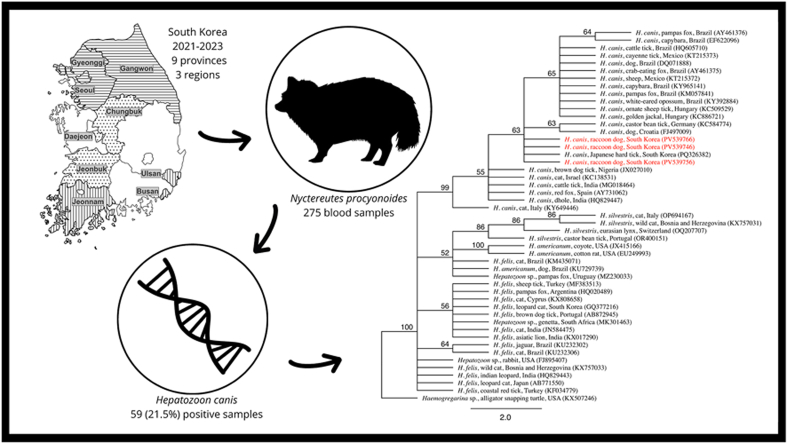

## 1 Introduction

The raccoon dog is a Canidae native to eastern Asia that is largely distributed in Europe as an introduced species (Ward and Wurster-Hill, 1990). Its role as an important carrier of tick-borne and zoonotic diseases has been well documented in both native and introduced habitats. Several publications have described the prevalence of protozoal, bacterial, and viral infections in raccoon dogs, as well as endo- and ectoparasitic infestations (Eo et al., 2012; Hwang et al., 2017; Kim et al., 2024; Myśliwy et al., 2022; Oba et al., 2025; Yang et al., 2021), the latter of which are responsible for vector-borne diseases that account for 17 % of all infectious diseases (World Health Organization, 2024).

Among the protozoan infections known to affect raccoon dogs, antibodies for *Neospora caninum* have been found in South Korea (Kim et al., 2003), and *Babesia vulpes* infections have also been described in South Korea (Han et al., 2010) and Austria (Duscher et al., 2017). One study on *Theileria* spp. was conducted in South Korea, but yielded no positive results (Han et al., 2017). Two recent studies investigated the presence of *Hepatozoon* spp. in introduced raccoon dogs but found no positive samples in both the Czech Republic and Austria (Daněk et al., 2023; Uiterwijk et al., 2023).

*Hepatozoon* is a genus of protozoa that parasitize the red and white blood cells of their intermediate hosts (vertebrates including amphibians, reptilians, avians, and mammals) and definitive hosts (hematophagous invertebrates) (Smith, 1996; Thomas et al., 2024). Among the over 300 species of *Hepatozoon* (Smith, 1996), *H. canis* is known to be well adapted to canid hosts (Baneth et al., 2003). In the Americas, nine out of ten canid species have been infected with *H. canis* (Thomas et al., 2024), and according to the National Center for Biotechnology Information (NCBI) database, there have also been records of canid species other than domestic dogs infected with *H. canis* in Africa, Asia, and Europe.

This study describes the first identification of *H. canis* in blood samples from raccoon dogs through molecular diagnosis, expanding the list of tick-borne diseases that can be carried throughout the environment. These findings emphasize the importance of monitoring the growing population of this species and its health in both native and introduced habitats.

## 2 Material and methods

### 2.1 Sample collection

Between 2021 and 2023, blood samples were collected from 275 raccoon dogs admitted to 9 wildlife centers across South Korea. The centers were divided into three regions according to their provincial limits as northern (Gangwon, Gyeonggi, and Seoul), central (Daejeon, Chungbuk, and Jeonbuk), and southern (Busan, Ulsan, and Jeonnam). The whole blood samples were collected into EDTA tubes and stored at −20 °C.

### 2.2 DNA extraction and PCR

DNA extraction was performed using the DNeasy Blood & Tissue Kit (Qiagen, Hilden, Germany) according to the manufacturer's instructions and stored at −20 °C. Conventional PCR was performed using a partial 18S rRNA gene sequence of *Hepatozoon* spp. The primer pair consisted of HepF (5-ATA-CAT-GAG-CAA-AAT-CTC-AAC-3) and HepR (5-CTT-ATT-ATT-CCA-TGC-TGC-AG-3) with a target of 660 base pairs. PCR was performed under the following conditions: an initial denaturation step of 95 °C for 5 min, 34 cycles consisting of denaturation at 95 °C for 30 s, annealing at 57 °C for 30 s, extension at 72 °C for 90 s, and a final extension step at 72 °C for 5 min (Inokuma et al., 2002). The products were run on 1.5 % agarose gel, and the results were visualized using UV light.

### 2.3 Nucleotide sequencing and phylogenetic analysis

PCR products were sent to SolGent (Korea) for nucleotide sequencing. The BLAST tool from the NCBI database was used to investigate the homology between the obtained sequence with those in the database. Sequences were aligned using the MAFFT algorithm implemented in Geneious Prime (version 2025.1.2; Biomatters Ltd.). Phylogenetic relationships were inferred using the Geneious Tree Builder, employing the Neighbor-Joining method with the Tamura-Nei genetic distance model. Node support was assessed using bootstrap resampling with 1000 replicates. The resulting tree was visualized and edited within Geneious Prime, and the final topology was rooted using *Haemogregarina* sp.

## 3 Results

**Fig. 1:**
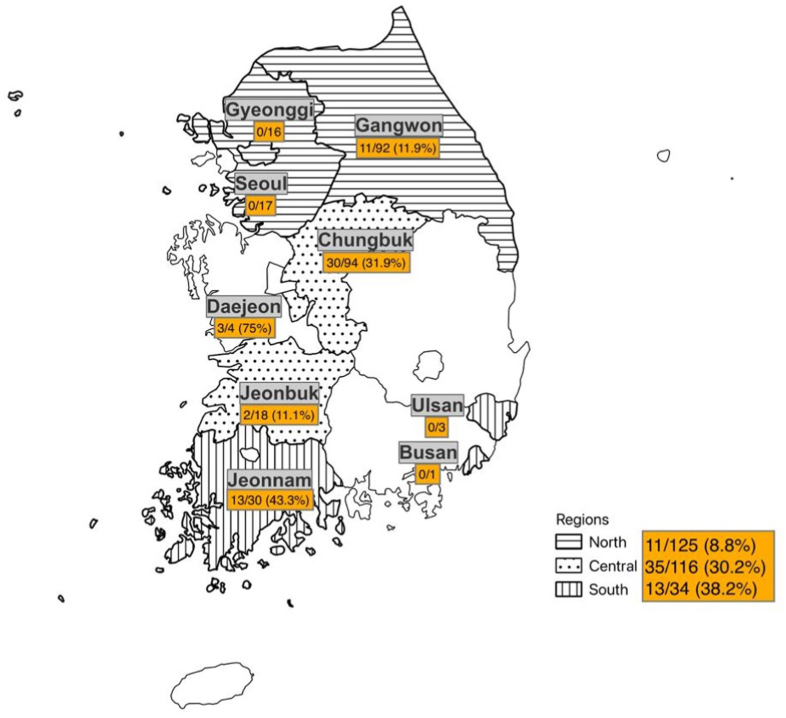
Geographic distribution and prevalence of *H. canis* in raccoon dogs admitted to wildlife centers across South Korea (2021–2023). Prevalence is shown as the number of positive cases over the total number of individuals tested per province with percentages in parentheses. Provinces were grouped into three regions: north (striped), central (dotted), and south (vertical lines).

A total of 275 blood samples from raccoon dogs from 9 wildlife centers across South Korea were analyzed. The samples were distributed as follows: 125 from the northern region (Gangwon: 92, Gyeonggi: 16, and Seoul: 17), 116 from the central region (Daejeon: 4, Chungbuk: 94, and Jeonbuk: 18), and 34 from the southern region (Busan: 1, Ulsan: 3, and Jeonnam: 30) (Fig. 1).

**Fig. 2:**
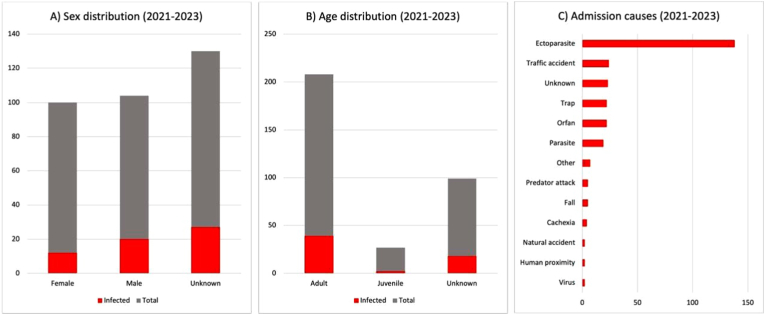
Sex, age, and admission causes of raccoon dogs sampled between 2021 and 2023. (A) Sex distribution and infection prevalence. (B) Age distribution and infection prevalence. (C) Main causes of admission.

Sex was determined in 172 animals, with 88 females and 84 males (Fig. 2-A). Infection prevalence was higher in males (33.9 %) than in females (20.3 %). Age was determined in 194 individuals, with 169 adults and 25 juveniles (Fig. 2-B). Infection was more frequent in adults (66.1 %) than in juveniles (3.4 %). The leading cause of admission was ectoparasite infestation (50.2 %), followed by traffic accidents (8.7 %), unknown causes (8.4 %), and trapping (8 %), whereas other causes such as orphaning, parasitic disease, predation, accidents, cachexia, human proximity, and viral infection were less frequent (Fig. 2-C).

Overall, the prevalence of *H. canis* among the 275 samples was 21.5 %. Among the nine sampled areas, four did not have any positive samples. The area with the highest prevalence was Daejeon (75 %), and the lowest was Jeonbuk (11.1 %). With regard to the different regions analyzed, the southern region (38.2 %) had the highest prevalence, and the northern region (8.8 %) had the lowest prevalence (Table 1).

**Table 1:** Prevalence of *Hepatozoon canis* in raccoon dogs throughout South Korea from 2021 to 2023 divided by wildlife center and region.

Region	Center	Samples	Positive samples (infection rate [%])
			*H. canis*
Northern	Gangwon	92	11 (11.9)
	Gyeonggi	16	0
	Seoul	17	0
Subtotal	Northern	125	11 (8.8)

Central	Daejeon	4	3 (75)
	Chungbuk	94	30 (31.9)
	Jeonbuk	18	2 (11.1)
Subtotal	Central	116	35 (30.2)

Southern	Busan	1	0
	Ulsan	3	0
	Jeonnam	30	13 (43.3)
Subtotal	Southern	34	13 (38.2)

Total		275	59 (21.5)

**Fig. 3:**
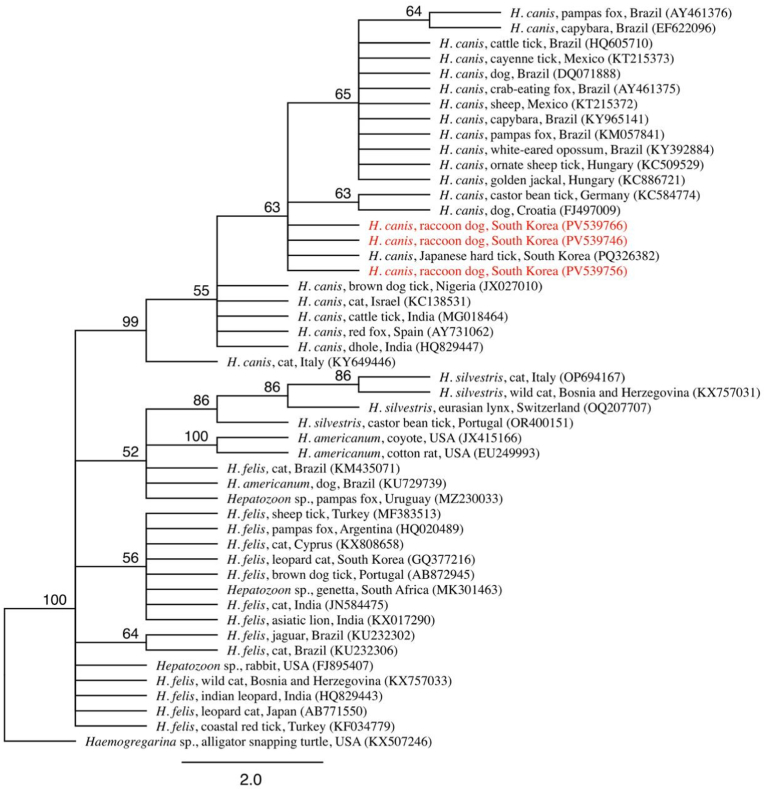
Phylogenetic relationships of *Hepatozoon* spp. based on 48 sequences (200 bp) of the 18S rRNA gene. The tree was constructed using the Geneious Prime (version 2025.1.2) tree builder, employing the Tamura-Nei model and the Neighbor-Joining method. Node support was evaluated by bootstrap analysis with 1000 replicates. Sequences obtained in this study are shown in red. *Haemogregarina* sp. was used as the outgroup. The scale bar represents phylogenetic distance.

The sequences obtained in this study were registered in the NCBI GenBank under accession numbers PV539747-PV539769. The sequences were 100 % identical between each other and showed a homogeneity of 97.7–100 % with other *H. canis* sequences deposited in the GenBank (Fig. 3). Phylogenetic analysis based on 18S rRNA gene sequences demonstrated that the *H. canis* isolates from raccoon dogs formed a distinct and strongly supported monophyletic clade within the broader a *H. canis* sequence found in a tick from South Korea (bootstrap value = 63). These raccoon dog-derived sequences clustered closely together and were clearly separated from *H. canis* isolates originating from other regions and host species, including domestic dogs and wild canids from Brazil, Europe, and Africa. No raccoon dog sequences clustered with non-*H. canis* taxa such as *H. felis*, *H. silvestris*, or *H. americanum*, confirming their species identity.

## 4 Discussion

Research on *H. canis* is mainly focused on domestic dogs worldwide, and the pathogen has been described as highly prevalent (Hasani et al., 2024). However, published articles on the topic in South Korea are limited to a few studies. The first description of *H. canis* in a Korean domestic dog was a case report from 2017 (Kwon et al., 2017), followed by three surveys with large sample sizes of 440, 510, and 906 animals, finding 1 (0.2 %), 2 (0.4 %), and 9 (0.9 %) positive samples, respectively (Kim and Seo, 2023; Seo et al., 2020; Suh et al., 2017). The prevalence of 0.2–0.9 % of *H. canis* in Korean domestic dogs shows considerable contrast with the overall prevalence of 21.5 % in raccoon dogs found in the present study. A similar scenario has been described between domestic dogs and red foxes (*Vulpes vulpes*) coexisting in the same geographic location, where dogs had a significantly lower infection rate than red foxes, which are considered the primary reservoir hosts (Cassini et al., 2009; Gabrielli et al., 2010). However, being an enzootic infection, numerous publications about hepatozoonosis in domestic and wild canids have shown different infection rates across different regions (Thomas et al., 2024; Uiterwijk et al., 2023), reinforcing the need for a systematic approach involving direct and indirect studies on this parasite and its intermediary and definitive hosts (Hasani et al., 2024).

Furthermore, from the three studies surveying *H. canis* in domestic dogs in South Korea, one identified the highest prevalence in the country's northern region, while the other two found it to be in the southern region, similar to the present study. In addition, recent research on tick-borne protists in ticks from South Korea found a single male Japanese hard tick (*Ixodes nipponensis*) (0.09 %) infected with *H. canis* among over 13,000 specimens from 5 different tick species (Alkathiri et al., 2024). The positive sample was found in the country's central region and represents the first description of *H. canis* in a tick from South Korea. The study suggests that Japanese hard ticks may be possible vectors of *H. canis* since South Korea is not an endemic region to brown dog ticks (*Rhipicephalus sanguineus*), which are the main known vectors of *H. canis* (Alkathiri and Lee, 2022).

The lack of studies on the presence of *Hepatozoon* species in wild animals from South Korea makes it difficult to discuss and trace the epizootiology of this pathogen. The low prevalence of *H. canis* in Korean domestic dogs and tick populations is dissonant from the prevalence observed in the raccoon dogs sampled in the present study, which may suggest another hypothesis for the seemingly high infection rate of *H. canis* in raccoon dogs: transplacental transmission of type 2 merozoites has been confirmed to occur in *H. canis* in dogs and red foxes (Hodžić et al., 2018; Murata et al., 1993), and thus could also be a possibility for raccoon dogs. Furthermore, infection with *H. americanum* has been described secondary to the consumption of infected prey tissue carrying meronts or cystozoites (Eileen M. Johnson et al., 2009a; E. M. Johnson et al., 2009b). Although this route of transmission has yet to be described for *H. canis,* the possibility of consumption of *H. canis*-infected ticks attached to the prey should not be discarded, which would make raccoon dogs more susceptible to infection than domestic dogs since these wild canids prey on small mammals while domestic dogs rarely or never do. Therefore, studies on the prevalence of *H. canis* in raccoon dogs’ prey and their ticks should be encouraged.

To the best of our knowledge, *Hepatozoon* infection in South Korea has previously been reported only in domestic dogs, a leopard cat (*Prionailurus bengalensis*) (Kubo et al., 2010), a yellow-throated marten (*Martes flavigula*) (Park et al., 2016), and one Japanese hard tick. Among these, the tick-derived isolate showed 100 % identity with the sequences obtained in the present study. This finding may suggest the presence of a geographically distinct *Hepatozoon* lineage. However, further research involving additional potential hosts is necessary to better elucidate the genetic diversity and transmission dynamics of this parasite.

## 5 Conclusions

This study reports the first detection of *H. canis* in raccoon dogs in South Korea and globally, significantly expanding the known host range of this parasite. The high infection prevalence observed suggests that raccoon dogs may serve as important reservoirs for *H. canis* within native ecosystems. Given the low prevalence in domestic dogs and ticks reported in previous studies, alternative transmission pathways including predation or vertical transmission warrant further investigation. Monitoring the health status of raccoon dogs is essential not only for wildlife conservation but also for public and animal health under the One Health approach. Continued research should explore the broader ecological impacts of *H. canis* infections in raccoon dogs and related species.

## CRediT authorship contribution statement

**Itainara Taili:** Writing – original draft, Software, Methodology, Formal analysis, Data curation, Conceptualization. **Jongseung Kim:** Writing – review & editing, Resources, Data curation. **Sungryong Kim:** Writing – review & editing, Validation, Investigation, Data curation. **Dong-Hyuk Jeong:** Writing – review & editing, Writing – original draft, Resources, Data curation. **Ki-Jeong Na:** Writing – review & editing, Validation, Supervision, Project administration, Methodology, Investigation, Funding acquisition, Conceptualization.

## Conflict of interest

The authors declare no conflict of interest.

## References

[bib1] Alkathiri B., Lee S., Ahn K.S., Youn S.Y., Yoo M.S., Lee H.S., Cho Y.S., Jung J., Seo K., Kim S., Umemiya-Shirafuji R., Xuan X., Kwak D., Shin S.S., Lee S.H. (2024). DNA barcoding using 18S rRNA gene fragments for identification of tick-borne protists in ticks in the Republic of Korea. Pathogens.

[bib2] Baneth G., Mathew J.S., Shkap V., Macintire D.K., Barta J.R., Ewing S.A. (2003). Canine hepatozoonosis: two disease syndromes caused by separate hepatozoon spp. Trends Parasitol..

[bib3] Cassini R., Zanutto S., di Regalbono A.F., Gabrielli S., Calderini P., Moretti A., Tampieri M.P., Pietrobelli M. (2009). Canine piroplasmosis in Italy: epidemiological aspects in vertebrate and invertebrate hosts. Vet. Parasitol..

[bib4] Daněk O., Lesiczka P.M., Hammerbauerova I., Volfova K., Juránková J., Frgelecová L., Modrý D., Hrazdilova K. (2023). Role of invasive carnivores (*Procyon lotor* and *Nyctereutes procyonoides*) in epidemiology of vector-borne pathogens: molecular survey from the Czech Republic. Parasites Vectors.

[bib5] Duscher T., Hodžić A., Glawischnig W., Duscher G.G. (2017). The raccoon dog (*Nyctereutes procyonoides*) and the raccoon (*procyon lotor*)-their role and impact of maintaining and transmitting zoonotic diseases in Austria, central Europe. Parasitol. Res..

[bib6] Eo K.Y., Kwak D., Kwon O.D. (2012). Detection of gastrointestinal parasites in raccoon dogs (*Nyctereutes procyonoides*) in the seosan reclaimed lands. Korea. J. Zoo Wildl. Med..

[bib7] Gabrielli S., Kumlien S., Calderini P., Brozzi A., Iori A., Cancrini G. (2010). The first report of hepatozoon canis identified in *vulpes vulpes* and ticks from Italy. Vector Borne Zoonotic Dis..

[bib8] Han J.I., Lee S.J., Jang H.J., Na K.J. (2010). Asymptomatic *babesia microti*-like parasite infection in wild raccoon dogs (*Nyctereutes procyonoides*) in South Korea. J. Wildl. Dis..

[bib9] Han Y.J., Park J., Lee Y.S., Chae J.S., Yu D.H., Park B.K., Kim H.C., Choi K.S. (2017). Molecular identification of selected tick-borne pathogens in wild deer and raccoon dogs from the Republic of Korea. Vet. Parasitol. Reg. Stud. Reports.

[bib10] Hasani S.J., Rakhshanpour A., Enferadi A., Sarani S., Samiei A., Esmaeilnejad B. (2024). A review of hepatozoonosis caused by *hepatozoon canis* in dogs. J. Parasit. Dis..

[bib11] Hodžić A., Alić A., Duscher G.G. (2018). High diversity of blood-associated parasites and bacteria in European wild cats in Bosnia and Herzegovina: a molecular study. Ticks Tick Borne Dis..

[bib12] Hwang J., Lee K., Kim Y.J., Sleeman J.M., Lee H. (2017). Retrospective analysis of the epidemiologic literature, 1990-2015, on wildlife-associated diseases from the Republic of Korea. J. Wildl. Dis..

[bib13] Inokuma H., Okuda M., Ohno K., Shimoda K., Onishi T. (2002). Analysis of the 18S rRNA gene sequence of a Hepatozoon detected in two Japanese dogs. Vet. Parasitol..

[bib14] Johnson Eileen M., Allen K.E., Panciera R.J., Ewing S.A., Little S.E. (2009). Experimental transmission of *Hepatozoon americanum* to New Zealand white rabbits (*oryctolagus cuniculus*) and infectivity of cystozoites for a dog. Vet. Parasitol..

[bib15] Johnson E.M., Panciera R.J., Allen K.E., Sheets M.E., Beal J.D., Ewing S.A., Little S.E. (2009). Alternate pathway of infection with *Hepatozoon americanum* and the epidemiologic importance of predation. J. Vet. Intern. Med..

[bib16] Kim J.H., Kang M.S., Lee B.C., Hwang W.S., Lee C.W., So B.J., Dubey J.P., Kim D.Y. (2003). Seroprevalence of antibodies to *Neospora caninum* in dogs and raccoon dogs in Korea. Kor. J. Parasitol..

[bib17] Kim K.T., Seo M.G. (2023). Molecular analysis of *rickettsia* spp. and related tick-borne pathogens detected in dogs in Korea. Acta Trop..

[bib18] Kim M., Win P.W., Kim Y.H., Han J.I. (2024). Successful treatment of scabies-induced life threatening anemia in a wild raccoon dog (*Nyctereutes procyonoides*). J. Vet. Clin..

[bib19] Kubo M., Jeong A., Kim S.I., Kim Y.J., Lee H., Kimura J., Agatsuma T., Sakai H., Yanai T. (2010). The first report of hepatozoon species infection in leopard cats (*Prionailurus bengalensis*) in Korea. J. Parasitol..

[bib20] Kwon S.J., Kim Y.H., Oh H.H., Choi U.S. (2017). First case of canine infection with *hepatozoon canis* (apicomplexa: Haemogregarinidae) in the Republic of Korea. Kor. J. Parasitol..

[bib21] Murata T., Inoue M., Tateyama S., Taura Y., Nakama S. (1993). Vertical transmission of *hepatozoon canis* in dogs. J. Vet. Med. Sci..

[bib22] Myśliwy I., Perec-Matysiak A., Hildebrand J. (2022). Invasive raccoon (*Procyon lotor*) and raccoon dog (*Nyctereutes procyonoides*) as potential reservoirs of tick-borne pathogens: data review from native and introduced areas. Parasites Vectors.

[bib23] Oba M., Sakaguchi S., Teshima N., Yokota T., Takemae H., Tohei M., Shimokawa F., Murakami M., Mizuno S., Ishida H., Murakami H., Takano T., Mizutani T., Tsukada H., Nagai M. (2025). Metatranscriptomic identification of novel RNA viruses from raccoon dog (*Nyctereutes procyonoides*) feces in Japan. Sci. Rep..

[bib24] Park S., Choi U.S., Kim E.J., Lee J.H., Lee H.B., Cho H.S., Kim W., Lim C.W., Kim B. (2016). Coinfection with *Hepatozoon* sp. and canine distemper virus in a yellow-throated marten (*Martes flavigula koreana*) in Korea. J. Wildl. Dis..

[bib25] Seo M.G., Kwon O.D., Kwak D. (2020). Molecular detection and phylogenetic analysis of canine tick-borne pathogens from Korea. Ticks Tick Borne Dis.

[bib26] Smith T.G. (1996). The genus hepatozoon (apicomplexa: adeleina). Source. J. Parasitol..

[bib27] Suh G.H., Ahn K.S., Ahn J.H., Kim H.J., Leutenegger C., Shin S.S. (2017). Serological and molecular prevalence of canine vector-borne diseases (CVBDs) in Korea. Parasites Vectors.

[bib28] Thomas R., Santodomingo A., Saboya-Acosta L., Quintero-Galvis J.F., Moreno L., Uribe J.E., Muñoz-Leal S. (2024). Hepatozoon (eucoccidiorida: hepatozoidae) in wild mammals of the americas: a systematic review. Parasites Vectors.

[bib29] Uiterwijk M., Vojta L., Šprem N., Beck A., Jurković D., Kik M., Duscher G.G., Hodžić A., Reljić S., Sprong H., Beck R. (2023). Diversity of hepatozoon species in wild mammals and ticks in Europe. Parasites Vectors.

[bib30] Ward O., Wurster-Hill D. (1990). Nytereutes procyonoides. Mamm. Spec..

[bib31] World Health Organization (2024). Vector-borne diseases. https://www.who.int/news-room/fact-sheets/detail/vector-borne-diseases.

[bib32] Yang S., He Y., Chen X., Kalim U., Wang Y., Yang S., Qi H., Cheng H.Z., Lu X., Wang X., Shen Q., Zhang W. (2021). Viral metagenomics reveals diverse viruses in the feces samples of raccoon dogs. Front. Vet. Sci..

